# Differentiation of Motor Neuron-Like Cells from Tonsil-Derived Mesenchymal Stem Cells and Their Possible Application to Neuromuscular Junction Formation

**DOI:** 10.3390/ijms20112702

**Published:** 2019-06-01

**Authors:** Saeyoung Park, Ji Yeon Kim, Seoha Myung, Namhee Jung, Yeonzi Choi, Sung-Chul Jung

**Affiliations:** Department of Biochemistry, College of Medicine, Ewha Womans University, Seoul 07804, Korea; saeyoung@ewha.ac.kr (S.P.); jiyeon12@ewha.ac.kr (J.Y.K.); myothink@naver.com (S.M.); prettycy@naver.com (N.J.); choiz@ewhain.net (Y.C.)

**Keywords:** tonsil-derived mesenchymal stem cells, neural precursor cell, motor neuron, neuromuscular junction, acetylcholine, motor neuron disease

## Abstract

Human tonsil-derived mesenchymal stem cells (T-MSCs) are newly identified MSCs and present typical features of MSCs, including having the differentiation capacity into the three germ layers and excellent proliferation capacity. They are easily sourced and are useful for stem cell therapy in various disease states. We previously reported that T-MSCs could be differentiated into skeletal myocytes and Schwann-like cells; therefore, they are a promising candidate for cell therapies for neuromuscular disease. Motor neurons (MNs), which regulate spontaneous behavior, are affected by a wide range of MN diseases (MNDs) for which there are no effective remedies. We investigated the differentiation potential of MN-like cells derived from T-MSCs (T-MSC-MNCs) for application to therapy of MNDs. After the process of MN differentiation, the expression of MN-related markers, including Islet 1, HB9/HLXB9 (HB9), and choline acetyltransferase (ChAT), was increased when compared with undifferentiated T-MSCs. The secretion of acetylcholine to the conditioned medium was significantly increased after MN differentiation. We cocultured T-MSC-MNCs and human skeletal muscle cells, and confirmed the presence of the acetylcholine receptor clusters, which demonstrated the formation of neuromuscular junctions. The potential functional improvements afforded by these T-MSC-MNCs could be useful in the treatment of MNDs caused by genetic mutation, viral infection, or environmental problems.

## 1. Introduction

Human tonsil-derived mesenchymal stem cells (T-MSCs) have typical features of MSCs that can differentiate into adipocytes, chondrocytes, and osteocytes, and that mediate immunomodulation [1,2,3]. T-MSCs are a useful source for cell therapy because they can be separated from human tonsil tissue that is discarded after surgery. They are affected by donor age and can be cultured long-term (15 passages) and cryopreserved while maintaining their morphology, cell surface markers, and differentiation potential [4,5]. T-MSCs have been reported to differentiate into the three primary germ layers, i.e., mesodermal lineage (including fat, cartilage, bone, and muscle cells), endodermal lineage (including the liver and insulin-secreting and parathyroid hormone-secreting cells), and ectodermal lineage (including Schwann and neuronal cells) [4,6,7,8,9,10,11,12].

A motor neuron (MN) sends signals from the brain to the muscles and bones to make the muscles move. There are two types of MN: upper and lower MNs. Lower MNs originate in the spinal cord and directly or indirectly innervate effector targets. One type of lower MN is the somatic MN, which projects its axons to skeletal muscles. MN diseases (MNDs) are a group of progressive neurological disorders that destroy MNs, including the cells that regulate essential spontaneous muscle activities, such as speaking, walking, breathing, and swallowing. Some MNDs are hereditary, and nonhereditary or sporadic MND are related to environmental, toxic, viral, or genetic factors [13,14].

Several studies have been performed using stem cells to treat MNDs including amyotrophic lateral sclerosis (ALS), primary lateral sclerosis, progressive muscular atrophy, and progressive bulbar palsy [15]. Studies on the differentiation of induced pluripotent stem cell (iPSC)-derived MNs (iPS-MNs) have been performed, and validation tests, such as transplantation into diseased animals, have been reported. Several studies have reported the differentiation of embryonic stem cells (ESCs) or iPSC into MNs for the treatment of MND [16,17,18,19]. Although ESCs/iPSCs have potential as MND therapeutics, they also have limitations, including tissue compatibility and tumor formation, and their source raises ethical concerns. Therefore, adult stem cells, such as MSCs, are a suitable source of cells for stem cell therapy and may be used to promote the neuromuscular regeneration of patients with MND. Several studies have reported that the MSCs from the umbilical cord, bone marrow, Wharton’s jelly, and dental pulp can differentiate into MNs [20,21,22,23]. However, there was no definite evidence that MSC-derived MNs is functionally active.

In the present study, we assessed a simple and rapid protocol for generating T-MSC-derived MN cells (T-MSC-MNCs) as a cell therapy source for MND. The T-MSC-MNCs were evaluated by quantitative reverse transcription polymerase chain reaction (RT–qPCR), western blot analysis, and immunocytochemistry. To confirm the efficiency of the functional differentiation of T-MSC-MNCs, we analyzed the change in acetylcholine secretion and the formation of acetylcholine receptor clusters in culture. We report for the first time that T-MSC can differentiate to MNs and describe the method for their differentiation using the process for differentiating neural progenitor cells (NPCs).

## 2. Results

### 2.1. Differentiation of MN-Like Cells from T-MSCs

**Figure 1:**
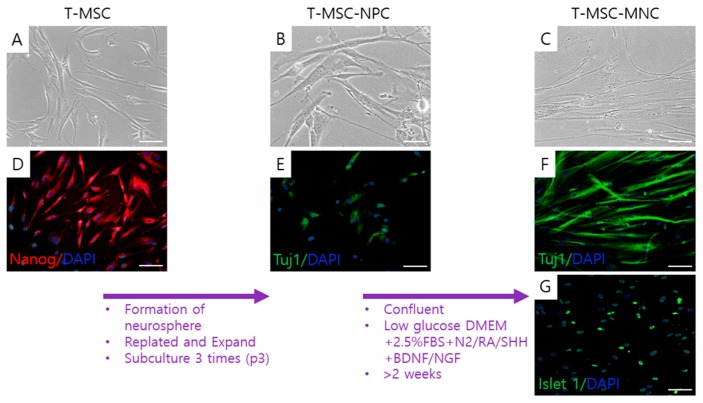
Schematic of differentiation of tonsil-derived mesenchymal stem cells (T-MSCs) toward motor neuron (MN)-like cells (MNCs). (**A**) The undifferentiated T-MSCs were induced to (**B**) T-MSC-derived neural precursor cells (T-MSC-NPCs). (**C**) T-MSC-MNCs were induced by culturing T-MSC-NPCs in MN differentiation medium for 14 days. Expression of pluripotent (**D**; Nanog; red), neuronal (**E**,**F**; Tuj1; green), motor neuronal markers (**G**; Islet 1; green) and total cell (**D**–**G**; DAPI; blue) was examined using immunocytochemistry in cells at each stage. Scale bars = 100 μm. Tuj1, β-III tubulin; DAPI, 4′,6-diamidino-2-phenylindole; RA, retinoic acid; Shh, sonic hedgehog; BDNF, brain-derived neurotrophic factor; NGF, nerve growth factor.

To induce differentiation to MNCs, the undifferentiated T-MSCs (Figure 1A) that expressed Nanog (Figure 1D) were differentiated to NPCs using a previously established method of formation of spheroids on a polyethyleneimine (PEI)-coated culture dish [24]. T-MSC-NPCs elongated with increasing size of the cells (Figure 1B) after the differentiation and weakly expressed Tuj1 (Figure 1E), a neuronal cell factor. Subsequently, the T-MSC-NPCs were differentiated into T-MSC-MNCs by culture in the motor neuronal induction medium (MNM). The cells became interconnected, exhibiting a neuronal-like morphology. After the final differentiation into the β-III tubulin (Tuj1)+/Islet 1+ T-MSC-MNCs (Figure 1F,G), the cells were thin, elongated, connected, and looked like nerve cells (Figure 1C).

### 2.2. Detection of Motor Neuronal Markers in T-MSC-MNCs

#### 2.2.1. RT–qPCR

**Figure 2:**
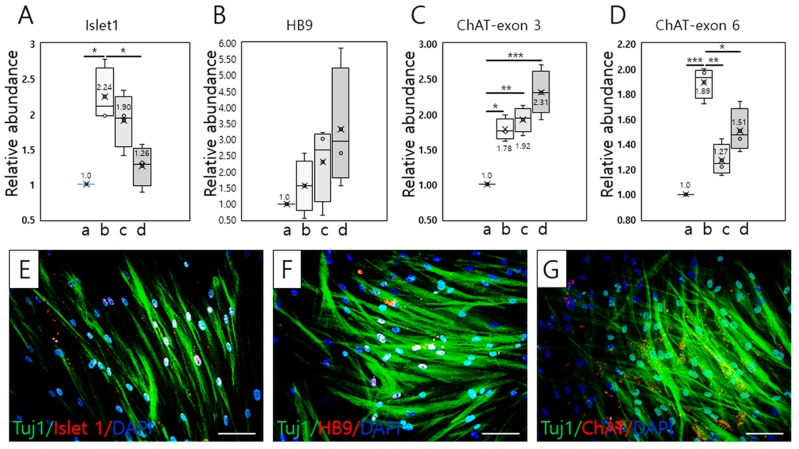
Schematic of the derivation of MN-like cells from T-MSCs. (**A**–**D**) Time-course RT–qPCR analysis of the expression of Islet 1, HB9, and ChAT during differentiation of T-MSC-MNCs (*n* = 3). Data are presented as the mean ± SEM of at least three experiments. *a* = T-MSC; *b* = 2 weeks differentiation; *c* = 3 weeks differentiation; *d* = 4 weeks differentiation. The statistical analysis was performed using one-way ANOVA (* *p* < 0.05; ** *p* < 0.01; ** *p* < 0.001). (**E**–**G**) Double immunostaining analysis for detection of MN markers, Islet 1 (**E**), HB9 (**F**), and ChAT (**G**) plus Tuj1 after 2 weeks of MN differentiation. DAPI staining (blue), immunostaining for neuron-specific β-tubulin class III (Tuj1; green), Islet 1 (red), homeobox HB9 (HB9; red). White indicates the merged images for DAPI, Tuj1, and Islet 1 or HB9; yellow indicates the merged images for Tuj1 and ChAT. Scale bars = 100 μm.

To investigate the effective period for differentiation of T-MSC-MNCs, we examined the temporal alterations in the expression of several MN-related genes (Islet 1, HB9/HLXB9 (HB9), and choline acetyltransferase (ChAT)) during 4 weeks of differentiation culture using RT–qPCR analysis. The expression of Islet 1 increased significantly during the second weeks of culture, followed by a gradual decrease over the subsequent 2 weeks (* *p* < 0.05; Figure 2A). As shown in Figure 2B, HB9 gene expression showed a gradual increase over 4 weeks, but significance was not recognized (*p* = 0.288; Figure 2B). Notably, expression of both ChAT exon 3 was significantly increased compared with that in the T-MSCs (* *p* < 0.05; ** *p* < 0.01; *** *p* < 0.001; Figure 2C), but the expression of ChAT exon 6 was significantly lower than that of ChAT exon 3 after 3 (** *p* < 0.01) and 4 weeks of differentiation (*p* < 0.05; Figure 2D; Supplemental data 1). Peripheral type ChAT (pChAT) is preferentially expressed in the peripheral nervous system (PNS) in humans, and results from exon skipping (exons 6–9) during posttranscriptional modification. For this reason, we confirmed ChAT expression using primers specific to the two isoforms (exons 3 and 6, respectively). Based on ChAT expression analysis, T-MSC-MNCs might belong to the PNS.

#### 2.2.2. Immunocytochemistry

Consistent with the above results, immunocytochemical analysis revealed the efficient induction of MNCs on day 14 of MN differentiation (Figure 2E–G). The proportions of Islet 1+, HB9+, ChAT+, and Tuj1+ T-MSC-MNCs were 23.68% ± 1.70%, 11.74% ± 1.70%, 11.72% ± 1.74%, and 67.20% ± 3.96% of total cells, respectively (*n* = 3). In addition, the expression ratios of Islet 1, HB9, and ChAT in Tuj1+ T-MSCs-MNCs, identified as neurons, were 39.29% ± 1.16%, 19.69% ± 4.0%, and 16.10% ± 3.42%, respectively (*n* = 3; Supplemental data 2).

#### 2.2.3. Western Blot Analysis

**Figure 3:**
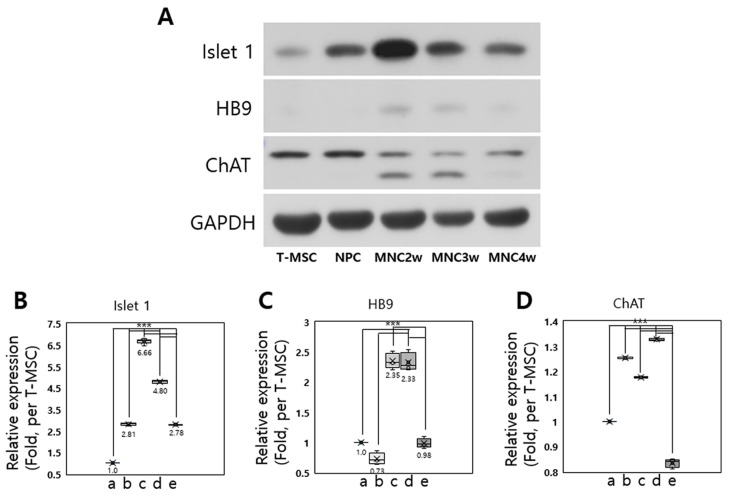
Detection of MN markers during differentiation into MN-like cells. (**A**) Western blot analysis of the expression of the Islet 1, HB9, and ChAT proteins during differentiation of T-MSC-MNCs from T-MSC (*n* = 3). The expression of the Islet 1 (**B**), HB9 (**C**), and ChAT (**D**) proteins was quantified by densitometry using ImageJ software. Protein expression levels are normalized to GAPDH. Data are presented as the mean ± SEM of at least three experiments. T-MSC (a); NPC (b) = neural precursor cell; MNC2w (c) = 2 weeks of differentiation; MNC3w (d) = 3 weeks of differentiation; and MNC4w (e) = 4 weeks of differentiation. The statistical analysis was performed using one-way ANOVA (*** *p* < 0.001).

We next examined the expression of several MN-related proteins during 4 weeks of differentiation into T-MSC-MNCs by western blot analysis (Figure 3A; Supplemental data 3). The expression of Islet 1 protein significantly increased during 4 weeks of differentiation (Figure 3B), and HB9 was higher in weeks 2 and 3 of MN differentiation than in undifferentiated T-MSCs, T-MSC-NPCs, and week 4 of MN differentiation (*p* < 0.001; Figure 3C). The expression of ChAT protein was increased slightly in T-MSC-NPC, and in weeks 2 and 3 of MN differentiation compared with that in T-MSCs, however it decreased in week 4 of MN differentiation (*p* < 0.001). Consistent with the characteristics of the ChAT gene expression described above, the 82- and 68-kDa ChAT isoforms were also detected in weeks 2 and 3 of MN differentiation (Figure 3D). Overall, these results confirmed that we had an efficient differentiation protocol that induced T-MSC-MNCs by differentiating T-MSC-NPCs for 2 weeks, even though we minimized the supplements in MNM and the required duration.

### 2.3. Effective MN Differentiation from T-MSCs

**Figure 4:**
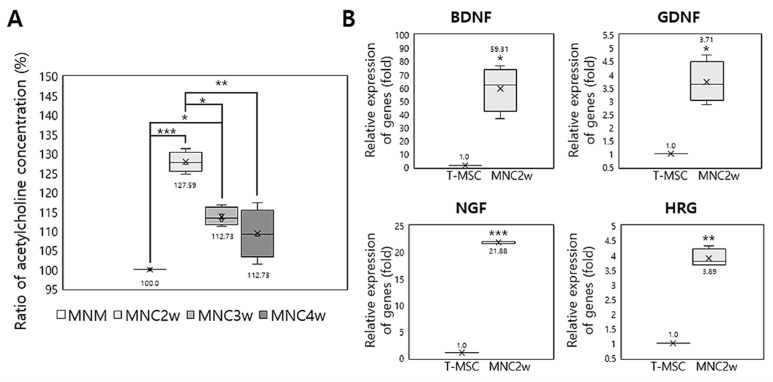
Changes in the secretion of functional MN-related factors after the differentiation of T-MSCs into T-MSC-MNCs. (**A**) Relative acetylcholine secretion during the differentiation into MNCs (*n* = 3). (**B**) Relative expression of neurotrophic factors by T-MSCs-MNCs assessed by RT–qPCR (*n* = 3). GAPDH served as a loading control. Data are expressed as the mean ± SEM. Triplicate independent mRNA samples were used in the RT–qPCR experiment. MNC1w = 1 week of differentiation; MNC2w = 2 weeks differentiation; MNC3w = 3 weeks differentiation; and MNC4w = 4 weeks differentiation. * *p* < 0.05; ** *p* < 0.01; *** *p* < 0.001.

The results of the enzyme-driven reaction assay for acetylcholine revealed that the secretion of acetylcholine was significantly increased in T-MSC-MNCs at 1, 2, 3, and 4 weeks (MN1w, MN2w, MN3w, and MN4w, respectively) following differentiation compared with that in fresh MNM (Figure 4A; Supplemental data 4). The ratios of the acetylcholine concentration compared with that in the MNM, defined as 100%, were 127.9% ± 1.925% (after 2 weeks differentiation; *p* < 0.001), 114.0% ± 1.592% (after 3 weeks differentiation; *p* < 0.05), and 109.4% ± 4.612% (after 4 weeks differentiation; *p* > 0.05). Notably, the observation that the concentration ratio was highest after 2 weeks differentiation confirmed the results above (Figure 2 and Figure 3), indicating that 2 weeks was the optimal time for differentiation. These results indicate that T-MSC-MNCs exhibit characteristics of cholinergic neuron.

### 2.4. Expression of Neurotrophic Factors by T-MSC-MNCs

To investigate whether T-MSC-MNCs showed enhanced expression levels of neurotrophic factors that promote the initial growth and development of neurons in the central nervous system (CNS) and PNS [25], we used RT–qPCR to analyze the expression of the neurotrophic factors (brain-derived neurotrophic factor (BDNF), glial cell-derived neurotrophic factor (GDNF), nerve growth factor (NGF), and heregulin (HRG)) in T-MSCs and T-MSC-MNCs (Figure 4B; Supplemental data 5). After MN differentiation of T-MSCs, the expression levels of BDNF (*p* < 0.05), GDNF (*p* < 0.05), NGF (*p* < 0.001), and HRG (*p* < 0.01) were significantly increased, especially those of BDNF and NGF.

### 2.5. Formation of Acetylcholine Receptor Clusters in Cocultures of T-MSC-MNCs with Human Skeletal Muscle Cells

**Figure 5:**
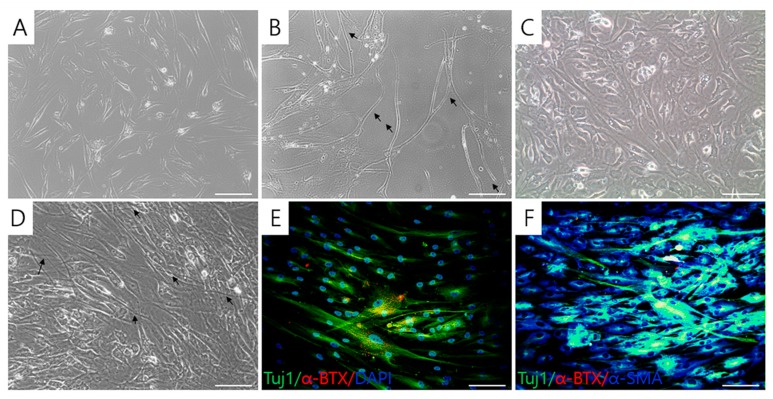
Formation of neuromuscular junctions in cocultures. Motor neuron-like cells (MNCs; **B**) derived from tonsil-derived mesenchymal stem cells (T-MSCs; **A**), and cocultured with human skeletal muscle cell (hSKMC)-derived myotubes (**C**) were observed under an inverted microscope (×200). After 14 days of coculture (**D**), aggregations of acetylcholine receptor (AchR) were detected by staining for DAPI (blue), immunostaining for β-III tubulin (Tuj1; green), α-smooth muscle actin (α-SMA; blue), and labelling with Alexa555-conjugated α-bungarotoxin (α-BTX; red) (**E**,**F**). Yellow fluorescence indicates the merged images for α-BTX and Tuj1, white fluorescence indicates the merged images of α-BTX, Tuj1, and α-SMA. Black arrows = T-MSC-MNC. Scale bars = 100 μm.

In addition to the above results, we also observed that the shape of T-MSC-MNCs differed from that of the undifferentiated T-MSC (Figure 5A). Compared with T-MSCs, T-MSC-MNCs became multipolar and the length of the cell body typically increased. They also had a bright nucleus and a cell body with a long axon-like structure ending in small extensions (black arrows; Figure 5B). To assess the functional characteristics of the T-MSC-MNCs, they were cocultured with human skeletal muscle cells (hSKMCs)-derived myotubes for 4 days (Figure 5C). After the coculture (Figure 5D), there was clustering of acetylcholine receptors (AchR) in the T-MSC-MNCs that could be visualized using Alexa 555-conjugated α-BTX (red), and the AchR clusters were colocalized with Tuj1 (green) and α-smooth muscle actin (α-SMA, blue) (white and yellow fluorescence, respectively) in α-bungarotoxin (α-BTX)+ cells (Figure 5E,F). These results suggest that the T-MSC-MNCs efficiently form functional neuromuscular junctions (NMJs) in coculture with hSKMC-derived myotubes.

## 3. Discussion

To induce the development of T-MSC-MNCs, we used a two-step differentiation procedure, differentiating T-MSCs into NPCs and the MNCs using different materials and methods. This method of differentiating T-MSCs into T-MSC-MNCs via NPCs seems to be a factor in the successful generation of functional MNCs. A previous study showed that iPSCs could also be differentiated into MNs, but did not demonstrate increased acetylcholine secretion or formation of NMJs [26]. A comparison of the differentiation methods used in our study with previously reported methods suggests that most of the factors added to the differentiation culture, e.g., retinoic acid (RA) and sonic hedgehog (Shh), are common to the methods, although some additional factors are added. There have been efforts to simplify the recently reported protocol for differentiation of human iPSCs to MNs with respect to time and procedures and improve its efficiency. The differentiation period is usually around 4 weeks when the NPC induction stage is included; however, the differentiation efficiency varied slightly depending on the cell source used or the markers used for identification. The proportions of Islet 1+ and HB9+ cells, considered to be indicators of MN differentiation, were 23.68% and 11.74%, respectively, of T-MSC-MNCs in this study and 70–90% and 35–46% for the iPS-MNCs generated in the other studies [18,26,27]. These results indicate that T-MSC can be differentiated into MNs, although their differentiation efficiency is lower than that of iPSC.

ChAT is responsible for the synthesis of the acetylcholine that is produced in the body of the neuron and is transported to the nerve terminal, where its concentration is the highest of all the nerve terminal proteins. ChAT is encoded by the CHAT gene in humans [28]. We analyzed the expression of CHAT for 4 weeks during MN differentiation; expression of exon 3 gradually increased over 4 weeks, but expression of exon 6 increased up to 2 weeks and decreased from 3 weeks. ChAT exists in two isoforms: the common type of ChAT (cChAT) exists in both the CNS and in the PNS. pChAT, which is present preferentially in the PNS, is formed by exon skipping (exon 6–9) during posttranscriptional modification. Therefore, our results suggest that T-MSC-MNCs belong to the PNS based on their specific expression of ChAT exons [29,30].

Concurrently with the increase in CHAT expression of T-MSC-MNCs, acetylcholine secretion into the CM also increased, demonstrating that T-MSCs can be differentiated into cholinergic neurons including MNs. Increased secretion of acetylcholine has also been reported in MN-like cells derived from bone marrow MSC [21] and umbilical cord MSC [20]. However, the presence of acetylcholine in iPSC-MNCs was confirmed by demonstrating the formation of NMJ, or by demonstrating their function as advanced MNs, such as a postsynaptic response. Acetylcholine is a substance used by the nervous system to activate skeletal muscles that are directly controlled by MNs located in the spinal cord or PNS. The contact between MNs and muscle fibers forms a chemical synapse, called NMJ [31]. In particular, the observation that acetylcholine clusters similar to those previously only observed in NMJ formed by iPSC-MNCs were also observed when T-MSC-MNCs were cocultured with hSKMCs further confirms the differentiation of MNs derived from T-MSCs.

Several studies have shown that the function and maintenance of NMJ are impaired in the early stages of MNDs, such as ALS and spinal muscular atrophy [32]. NMJ disorders have also been reported to play a critical role in the development of age-related sarcopenia [33]. In the present study, we used an in vitro coculture of MNCs and SKMCs, which should be useful for studies of NMJ-related disease. The use of this type of coculture has been reported mainly in studies using human iPSC-MNCs for the development of MND therapeutics [34]. There is currently no cure for MND, but only medications that relieve the symptoms and supportive treatment that can improve the quality of life of the patients. Such supportive treatments for MND include riluzole, which has been approved for the treatment of ALS, nusinersen, which has been approved for the treatment of spinal muscular atrophy, free radical scavengers that delay disease progression, muscle relaxants, such as baclofen, tizanidine, and benzodiazepine, and botulinum toxin. Thus, research into the development of new drugs that focus on genetic mutations and factors that affect the onset of MNDs has focused on drug interventions, gene therapy, and stem cell therapy [35]. Stem cell therapy with ESCs, iPSCs, neural stem cells, MSCs, and olfactory ensheathing stem cells has been studied in vitro and in mouse models, but the engraftment of stem cells into MND patients for therapeutic benefit remains challenging [36]. Some countries offer therapeutic trials of iPSCs treatment of MNDs, like ALS, but the lack of definite efficacy and safety makes this unsuitable for clinical application. Based on the results of previous studies of stem cell therapies for treating MND, the T-MSC-MNCs developed in this study should be suitable as therapeutic agents because they are capable of allotransplantation, it is easier to obtain the raw materials, and they have superior proliferation compared with other types of MSCs [27].

It is also noteworthy that the expression of the neurotrophic factors was increased after differentiation into T-MSC-MNCs. The various types of neurotrophic factors that are essential for MN maturation have already been reported [18,27]. In this study, the increased expression of GDNF and HRG genes, plus the BDNF and NGF that were added to the differentiation media for MN differentiation, demonstrated the functional superiority of T-MSC-MNCs. BDNF protects neurons via the tyrosine receptor kinase B and promotes the growth of damaged nerves. It is also often used for the maturation of MNs differentiated from ESC and iPSC [25]. In comparison, NGF uses the tyrosine receptor kinase A, supports myelination, promotes neural differentiation, and has been associated with maturation and survival of nerves for PNS development [37]. GDNF is a small protein that strongly stimulates the survival of many kinds of neurons and sends signals through the GDNF family receptor α (GFRα), particularly GFRα1. The most striking feature of GDNF is its ability to support the survival of MNs [38]. Finally, HRG has been shown to be able to indirectly signal muscle cells through Schwann cells to regulate the formation of NMJs [39,40].

## 4. Material and Methods

### 4.1. Ethics Statement

The T-MSCs were obtained from patients undergoing tonsillectomy. All the experimental procedures used in this study were approved by the Institutional Review Board of Ewha Womans University Mokdong Hospital (Seoul, Republic of Korea, permit No. ECT-11-53-02; approval date: 22 September 2011). We obtained an informed written consent from all patients and/or their legal representatives before study start.

### 4.2. Preparation of T-MSCs and their Differentiation into MN-like Cells

T-MSCs were isolated and cultured from tonsils collected from patients (≤10 years), as previously described [2,3]. The tonsillar tissues were minced and digested in Dulbecco’s modified Eagle’s medium (DMEM; Invitrogen, Carlsbad, CA, USA) containing 210 U/mL collagenase type I (Invitrogen) and DNase (10 µg/mL; Sigma-Aldrich, St. Louis, MO, USA). After the digested cells had been filtrated through a cell strainer (BD Biosciences, San Jose, CA, USA), mononuclear cells were collected by Ficoll-Paque (GE Healthcare, Chicago, IL, USA) density gradient centrifugation and cultured for 48 h at 37 °C in DMEM-low glucose/10% fetal bovine serum (FBS; Invitrogen) and 1% penicillin/streptomycin (Sigma-Aldrich) in a humidified chamber under 5% CO_2_ in air. To generate NPCs, T-MSC were induced to form spheroids 100–400 µm in diameter on the PEI-coated dish for 2–3 days. The formed spheroids were then transferred to a fresh culture dish for expansion in 10% FBS-supplemented low-glucose DMEM medium. Cells in the spheroids grew out of the aggregates and formed a rosette-like spread. Once confluent, cells were subcultured and expanded for up to 3 passages. To induce MN differentiation, 1.5–2 × 10^4^/cm^2^ T-MSC-NPCs were placed into motor neuronal induction medium (MNM), which comprised low-glucose DMEM supplemented with 2.5% FBS, 1% N_2_ supplement (Gibco, Life Technologies, Burlington, ON, Canada), 1 µM retinoic acid RA (Sigma-Aldrich), 10 ng/mL BDNF (R&D Systems, Minneapolis, MN, USA), 10 ng/mL NGF (R&D Systems), and 0.1 ng/mL Shh (R&D Systems). The T-MSC-MNCs were differentiated for 2–4 weeks under these conditions, and then harvested for characterization.

### 4.3. RT–qPCR

Qiagen RNeasy Mini Kits (Qiagen, Hilden, Germany) were used for RNA extraction from cells. For complementary DNA (cDNA) synthesis, Superscript II (Invitrogen) and oligo(dT)20 primers were used at 42 °C for 1 h followed by incubation at 72 °C for 15 min. RT–qPCR was performed using SYBR Premix Ex Taq DNA polymerase (Takara Bio, Shiga, Japan) on an ABI 7500 fast real-time PCR system (Applied Biosystems, Foster City, CA, USA), as described previously, to confirm the relative levels of expression of genes in the T-MSCs and T-MSC-MNCs. For quantification of the expression of each candidate gene, the mRNA expression levels were normalized to the glyceraldehyde 3-phosphate dehydrogenase (GAPDH) mRNA levels. The comparative threshold cycle (Ct) method (∆∆*C*t) was used to analyze relative gene expression [41]. RT–qPCR was performed in triplicate and repeated three times. The sequences of forward and reverse primers used were as follows: Islet1 forward 5′-AGCAGCCCAATGACAAAACT-3′, reverse 5′-CTGAAAAATTGACCAGTTGCTG-3′; HB9, forward 5′-GTCCACCGCGGGCATGATCC-3′, reverse 5′-TCTTCACCTGGGTCTCGGTGAGC-3′; ChAT exon 3, forward 5′-GGGCTGCCCAAACTGCCCG-3′, reverse 5′-CGAGACCCTGCAGCAGAAAC-3′; ChAT exon 2.3.1.6, forward 5′-TGTCTGAGTACTGGCTGAATGA-3′, reverse 5′-CACTTCCCTGGCACCGAT-3′; BDNF, forward 5′-GATGCCAGTTGCTTTGTCTTC-3′, reverse 5′-TAAAATCTCGTCTCCCCAACA-3′; GDNF, forward 5′-TTCAAGCCACCATTAAAAGAC-3′, reverse 5′-GACAAAGGTGTGAGTCGTGGT-3′; NGF, forward 5′–GTCAGCGTGTGGGTTGGGGATA–3′, reverse 5′–GACAAAGGTGTGAGTCGTGGT–3′; HRG, forward 5′–CGGTGTCCATGCCTTCCAT–3′, reverse 5′–GCGAGTTTCTTAACAGGCTCT-3′; GAPDH, forward 5′-ACACCCACTCCTCCACCTTT-3′, reverse 5′-TGCTGTAGCCAAATTCGTTG-3′.

### 4.4. Western Blotting

Protein samples from total cell extracts were washed with ice-cold phosphate-buffered solution (PBS). Protein samples were lysed in Pro-Prep buffer containing a phosphatase inhibitor cocktail solution (iNtRON Biotechnology, Seongnam-si, Korea) for 30 min on ice followed by centrifugation at 13,000× *g* for 20 min at 4 °C. Equal amounts of protein from each supernatant were separated by sodium dodecyl sulfate–polyacrylamide gel electrophoresis and were transferred onto polyvinylidene membranes (Millipore, Billerica, MA, USA). Then the blots were probed overnight at 4 °C with monoclonal antibodies against Islet 1 (Cat. no. ab86472), HB9 (Cat. no. ab79541), or ChAT (Cat. no. ab178850) (1:500, all from Abcam, Cambridge, UK) followed by the corresponding secondary antibody. The blots were developed using enhanced chemiluminescence reagents (WestSave Gold Western Blot Detection kits; AbFrontier, Seoul, Korea). The intensity of each band was assessed by densitometric scanning (LAS-3000; Fujifilm, Tokyo, Japan). The expression level of each protein was normalized with the respect of GAPDH levels (No. LF-PA0018; 1:1000, pRb, AbFrontier). The protein levels were quantified using Multi Gauge software (version 3.0; Fuji Photo Film, Kanagawa, Japan).

### 4.5. Immunocytochemistry

Cells were fixed in 4% (*v*/*v*) paraformaldehyde (Sigma-Aldrich) for 1 h at room temperature or overnight at 4 °C, then blocked in blocking buffer (PBS containing 2% bovine serum albumin (Bovogen Biologicals, East Keilor, Australia) in 0.1% Tween-20). Fixed cells were incubated in the diluted primary antibody for 1 h at room temperature or overnight at 4 °C. After washing three times in PBS, the samples were incubated for 1 h at room temperature with secondary antibodies diluted in PBS. Then the prepared samples were mounted using Vectashield mounting medium containing 4′,6-diamidino-2-phenylindole (DAPI; Vector Laboratories, Burlingame, CA, USA). Images were captured using a fluorescence microscope (Nikon, Tokyo, Japan). The antibodies used in the immunocytochemistry were as follows: mouse anti-Nanog (Cat. no. sc-374001; Santa Cruz Biotechnology, Santa Cruz, CA, USA), mouse anti-Tuj1 (Cat. no. ab14545; Abcam), rabbit anti-Tuj1 (Cat. no. ab52623; Abcam), mouse anti-Islet 1 (Cat. no. ab86472; Abcam), rabbit anti HB9 (Cat. no. ab79541; Abcam), rabbit anti-ChAT (Cat. no. ab178850; Abcam), mouse anti α-SMA (Cat. no. A2547; Sigma-Aldrich) (primary antibodies), and Alexa-568 goat anti-mouse IgG (Cat. no. A-11031), Alexa-488 goat anti-mouse IgG (Cat. no. A-11001), Alexa 568 goat anti-rabbit IgG (Cat. no. A-11011), Alexa-488 goat anti-rabbit IgG (Cat. no. A-11034), and Alexa-350 goat anti-mouse IgG (Cat. no. A-11045) (all from Life Technologies) (secondary antibodies). To stain AchR during neuromuscular junction development, Alexa Fluor 555-conjugated α-BTX was added to samples and incubated for 1 h at room temperature.

### 4.6. Enzyme-Driven Reaction Assay for Acetylcholine

To detect functional MN activity, we measured the acetylcholine content in the MNM and conditioned media (CM) after 1, 2, 3, and 4 weeks of MN differentiation. Samples were collected and stored at −80 °C. The CM sample was prepared by aspirating the culture medium and washing the cells twice with PBS, and then the T-MSC-MNCs were incubated with serum-free DMEM/F12 media for 24 h. The serum-free media was collected by centrifugation 1000 *g* for 5 min, and the supernatant collected as CM. Acetylcholine content was measured using an assay kit (Cell Biolabs, San Diego, CA, USA). A standard curve was used to determine the acetylcholine concentration in the tested samples. Results were measured using a microplate reader (Bio Tek Instruments, Winooski, VT, USA) as per the manufacturer’s instructions [42].

### 4.7. Coculture of Human Skeletal Muscle Cells with T-MSC-MNCs

Human skeletal muscle cells (hSKMC; Lonza Walkersville, Walkersville, MD, USA) were plated on cover glasses (Paul Marienfeld, Lauda-Kӧnigshofen, Germany) and cultured until the cells reached approximately 60% confluence, and then cultured in SKMC differentiation medium (PromoCell, Heidelberg, Germany) to induce the formation of myotubes. Subsequently, the medium was changed to MNM and the SKMC myotubes were cultured for 4 additional days. Finally, the T-MSC-MNCs were plated onto the SKMC-derived myotubes and cocultured for 3–4 days in MNM.

### 4.8. Statistical Analysis

Data are presented as the mean ± standard error of the mean (SEM) and analyzed using the GraphPad Prism version 4 (GraphPad Software, San Diego, CA, USA). Statistical comparisons between the control and test groups were analyzed by one-way analysis of variance (ANOVA) followed by Tukey’s post-hoc test for the western blotting, RT–qPCR and enzyme-driven reaction assay. Student’s *t*-test was used for the comparison of neurotrophic factors. Differences were considered significant at *p* < 0.05.

## 5. Conclusions

In conclusion, this study showed that we could differentiate T-MSC-NPCs derived from human T-MSCs into functional T-MSC-MNCs using our methods. We confirmed that expression of MN-related markers such as Islet 1, HB9, and ChAT by T-MSC-MNCs increased after MN differentiation compared with that in T-MSC. Also, T-MSC-MNCs showed increased acetylcholine secretion to the differentiating medium and acetylcholine receptor clusters were identified when they were cocultured with hSKMCs. These results indicate that the differentiation method used in this study was very effective. However, it is yet to be determined whether the functional MN differentiation and the morphological improvements in MNCs will be useful for the treatment of human MND and other neuromuscular disorders.

## Supplementary Materials

Supplementary materials can be found at https://www.mdpi.com/1422-0067/20/11/2702/s1.

## Abbreviations

T-MSC	tonsil-derived mesenchymal stem cell
MN	motor neuron
MND	motor neuron diseases
ALS	amyotrophic lateral sclerosis
ESC	embryonic stem cell
iPSC	induced pluripotent stem cell
NPC	neural progenitor cell
PEI	polyethyleneimine
PNS	peripheral nervous system
CNS	central nervous system
HB9	HB9/HLXB9
ChAT	choline acetyltransferase
pChAT	Peripheral type ChAT
MNM	motor neuronal induction medium
BDNF	brain-derived neurotrophic factor
GDNF	glial cell-derived neurotrophic factor
NGF	nerve growth factor
HRG	heregulin
hSKMC	human skeletal muscle cells
AchR	acetylcholine receptors
α-BTX	α-bungarotoxin
NMJs	neuromuscular junctions
RA	retinoic acid
Shh	sonic hedgehog
cChAT	common type of ChAT
GFRα	GDNF family receptor α
GAPDH	glyceraldehyde 3-phosphate dehydrogenase
FBS	fetal bovine serum
PBS	phosphate-buffered solution
CM	conditioned media
α-SMA	α-smooth muscle actin
SEM	standard error of the mean
ANOVA	analysis of variance
