# Supplementary material for: Differentiation of Motor Neuron-Like Cells from Tonsil-Derived Mesenchymal Stem Cells and Their Possible Application to Neuromuscular Junction Formation
Source: Int J Mol Sci. 2019 Jun 1;20(11):2702. doi: 10.3390/ijms20112702 (PMC6600529; doi:10.3390/ijms20112702)
Supplement: Supplementary file 1 [file ijms-20-02702-s001.pdf]

Supplemental data

Supplemental data 1: Raw data of the time-course RT–qPCR analysis of the expression of Islet 1, HB9, and ChAT during differentiation of T-MSC-MNCs (figure 2. A-D)

	Expression of gene	Sample	Experiment 1	Experiment 2	Experiment 3	Mean	SEM
A	Islet 1/GAPDH	a	1.00	1.00	1.00	1.00	0.00
		b	1.98	1.98	2.78	2.24	0.27
		c	1.41	2.33	1.97	1.90	0.27
		d	0.89	1.31	1.57	1.26	0.20
B	HB9/GAPDH	a	1.00	1.00	1.00	1.00	0.00
		b	0.54	1.56	2.57	1.56	0.59
		c	0.65	3.23	3.03	2.30	0.83
		d	2.58	1.55	5.85	3.33	1.30
	ChAT exon3/GAPDH	a	1.00	1.00	1.00	1.00	0.00
		b	1.61	1.74	1.98	1.78	0.11
		c	1.69	2.12	1.95	1.92	0.13
		d	2.30	1.92	2.70	2.31	0.23
	ChAT exon6/GAPDH	a	1.00	1.00	1.00	1.00	0.00
		b	2.00	1.97	1.72	1.89	0.09
		c	1.22	1.44	1.15	1.27	0.09
		d	1.74	1.44	1.34	1.51	0.12

a: T-MSC, b: 2 weeks of differentiation, c: 3 weeks of differentiation, d: 4 weeks of differentiation.

Supplemental data 2: Raw data of the immunostaining analysis for detection of MN markers, Islet 1, HB9, and ChAT plus Tuj1 after 2 weeks of MN differentiation (figure 2. E-G).

Figure 2	Expression of protein	Experiment 1	Experiment 2	Experiment 3	Mean (%)	SEM
E	Islet1/DAPI	20.37%	26.03%	24.64%	23.68	1.70
	Islet1/Tuj1	39.29%	41.30%	37.28%	39.29	1.16
F	HB9/DAPI	14.08%	12.70%	8.43%	11.74	1.70
	HB9/Tuj1	22.22%	25.00%	11.86%	19.69	4.00
G	ChAT/DAPI	12.70%	14.12%	8.33%	11.72	1.74

	ChAT/Tuj1	15.69%	22.22%	10.39%	16.10	3.42
E-G	Tuj1/DAPI	64.96%	61.75%	74.90%	67.2	3.96

Supplemental data 3: Raw data of the western blot analysis of the expression of the Islet 1, HB9, and ChAT proteins during differentiation of T-MSC-MNCs (figure 3. B-D).

Figure 2	Expression of Protein		Experiment 1	Experiment 2	Experiment 3	Mean	SEM
B	Islet 1/GAPDH	a	1	1	1	1.00	0.00
		b	2.84	2.86	2.74	2.81	0.04
		c	6.72	6.8	6.45	6.66	0.11
		d	4.85	4.84	4.7	4.80	0.05
		e	2.82	2.75	2.78	2.78	0.02
C	HB9/GAPDH	a	1	1	1	1	0
		b	0.86	0.69	0.63	0.73	0.07
		c	2.52	2.21	2.32	2.35	0.09
		d	2.54	2.21	2.24	2.33	0.11
		e	1.09	0.96	0.9	0.98	0.06
D	ChAT/GAPDH	a	1.0000	1.0000	1.0000	1.00	0.00
		b	1.2491	1.2568	1.2579	1.25	0.00
		c	1.1751	1.1789	1.1739	1.18	0.00
		d	1.3240	1.3242	1.3350	1.33	0.00
		e	0.8487	0.8107	0.8461	0.84	0.01

a: T-MSC, b: neural precursor cell, c: 2 weeks of differentiation, d: 3 weeks of differentiation, e: 4 weeks of differentiation.

Supplemental data 4: Raw data of the relative acetylcholine secretion during the differentiation into MNCs (figure 4. A).

	Experiment 1	Experiment 2	Experiment 3	Mean	SEM
MNM	100	100	100	100	0
MN2w	131.26	124.59	127.93	127.59	1.95
MN3w	116.78	111.27	114.02	112.73	2.05
MN4w	117.38	101.40	109.39	109.03	4.63

MNM: motor neuronal induction medium, MN2w: 2 weeks of differentiation, MN3w: 3 weeks of differentiation, MN4w: 4 weeks of differentiation

Supplemental data 5: Raw data of the relative expression of neurotrophic factors by T-MSCs-MNCs assessed by RT–qPCR (figure 4. B).

		Experiment 1	Experiment 2	Experiment 3	Mean	SEM
BDNF	T-MSC	1.00	1.00	1.00	1.00	0.00
	MNC2w	64.93	76.37	36.63	59.31	11.81
GDNF	T-MSC	1.00	1.00	1.00	1.00	0.00
	MNC2w	3.56	4.73	2.85	3.71	0.55
NGF	T-MSC	1.00	1.00	1.00	1.00	0.00
	MNC2w	21.54	22.05	22.05	21.88	0.17
HRG	T-MSC	1.00	1.00	1.00	1.00	0.00
	MNC2w	3.69	4.32	3.66	3.89	0.22
